# Altered Functional Connectivity and Sensory Processing in Blepharospasm and Hemifacial Spasm: Coexistence and Difference

**DOI:** 10.3389/fneur.2021.759869

**Published:** 2021-12-15

**Authors:** Ting-Chun Fang, Chun-Ming Chen, Ming-Hong Chang, Chen-Hao Wu, Yi-Jen Guo

**Affiliations:** ^1^Department of Neurology, Taichung Veterans General Hospital, Taichung City, Taiwan; ^2^Department of Medical Imaging, China Medical University Hospital, Taichung City, Taiwan; ^3^College of Life Science, National Chung Hsing University, Taichung City, Taiwan; ^4^Department of Radiology, Taichung Veterans General Hospital, Taichung City, Taiwan

**Keywords:** blepharospasm, hemifacial spasm, functional MRI, quantitative thermal test, resting state functional connectivity

## Abstract

**Background:** Blepharospasm (BSP) and hemifacial spasm (HFS) are both facial hyperkinesia however BSP is thought to be caused by maladaptation in multiple brain regions in contrast to the peripherally induced cause in HFS. Plausible coexisting pathophysiologies between these two distinct diseases have been proposed.

**Objectives:** In this study, we compared brain resting state functional connectivity (rsFC) and quantitative thermal test (QTT) results between patients with BSP, HFS and heathy controls (HCs).

**Methods:** This study enrolled 12 patients with BSP, 11 patients with HFS, and 15 HCs. All subjects received serial neuropsychiatric evaluations, questionnaires determining disease severity and functional impairment, QTT, and resting state functional MRI. Image data were acquired using seed-based analyses using the CONN toolbox.

**Results:** A higher cold detection threshold was found in the BSP and HFS patients compared to the HCs. The BSP and HFS patients had higher rsFC between the anterior cerebellum network and left occipital regions compared to the HCs. In all subjects, impaired cold detection threshold in the QTT of lower extremities had a correlation with higher rsFC between the anterior cerebellar network and left lingual gyrus. Compared to the HCs, increased rsFC in right postcentral gyrus in the BSP patients and decreased rsFC in the right amygdala and frontal orbital cortex in the HFS subjects were revealed when the anterior cerebellar network was used as seed.

**Conclusions:** Dysfunction of sensory processing detected by the QTT is found in the BSP and HSP patients. Altered functional connectivity between the anterior cerebellar network and left occipital region, especially the Brodmann area 19, may indicate the possibility of shared pathophysiology among BSP, HFS, and impaired cold detection threshold. Further large-scale longitudinal study is needed for testing this theory in the future.

## Introduction

Blepharospasm (BSP) is a focal dystonia characterized by bilateral, synchronous contractions of the orbicularis oculi. The etiology of BSP is unknown in most cases, but it may coexist with Parkinson's disease, progressive supranuclear palsy and multiple system atrophy, or it may be associated with lesions of the brainstem, thalamus, and basal ganglion (1). Neurophysiological and neuroimaging evidence suggests that BSP involves multiple brain regions including the basal ganglion, cerebellum, cerebral cortex, thalamus, and brainstem (2–5). Dysfunction within the sensorimotor system in BSP has been found. Overactivity of the postcentral gyrus and supplementary motor area were reported during whistling in BSP patients (6). One study showed reduced resting-state functional connectivity (rsFC) between sensorimotor cortex and several seeds including the caudate, cingulate gyrus, and cerebellum; besides, rsFC changes between the cerebellum and occipital cortex were also found, which suggest their possible functional role in sensory information or visuomotor integration in BSP (7). Defects in sensorimotor integration, loss of inhibition and maladaptive plasticity, may lead to BSP (2). Hemifacial spasm (HFS) is also a facial dyskinesia with unilateral paroxysmal contractions of facial muscles innervated by ipsilateral facial nerve. The most common etiology of HFS is compression at the root exit zone of facial nerve by aberrant vessels leading to spontaneous ephaptic nerve depolarizations in the root entry zone (8). HFS is considered to be a peripherally induced disorder; however, the antidromic transmission of stimuli may cause central and cortical reorganization (9, 10). In addition, atrophic amygdala and altered rsFC from the amygdala to the medial prefrontal cortex, orbital frontal cortex, and posterior insula have been reported, suggesting that the central mechanism of HFS may be associated with the disease severity and anxiety (11). Although BSP and HFS seem to be distinct diseases, a possible overlapping pathophysiologic mechanism is proposed (12). A study of structural brain networks also revealed increased global network segregation, decreased global efficiency and reduced degree of local structural network in the cerebella of both BSP and HFS patients (13).

Alterations of proprioceptive, tactile, nociceptive, and thermal information processing may be related to altered sensorimotor integration in focal dystonia, including blepharospasm (14). The somatosensory abnormalities are not only present in the body part affected by dystonia, but may also extend to the unaffected body parts (15–17). Quantitative thermal test (QTT) is a method to detect cold and heat detection thresholds. Previous study using quantitative sensory test reported impaired thermal detection threshold in patients with idiopathic focal dystonia (17).

Most previous functional studies have discussed BSP and HFS separately. In this study, we aimed to elucidate the correlations and differences of rsFC between BSP and HFS, by using known networks as seeds from the functional MRI (fMRI). We proposed that there might be altered connections between the cerebellum, sensorimotor cortex, and occipital lobe as the results reported by previous studies (6, 7, 13). We also hypothesized that there may be differences in sensory processing between patients with BSP, HFS, and healthy controls (HCs), which could be detected by the QTT.

## Materials and Methods

### Subjects

A total of 38 subjects were studied, including 12 patients with BSP (disease duration of 8.33 ± 5.30 years), 11 patients with HFS (disease duration of 8.50 ± 3.68 years), and 15 HCs. The patients with BSP and HFS were under regular treatment of local injection of botulinum toxin (BTX), with mean treatment duration of 3.83 ± 0.94 years in the BSP group, and 3.83 ± 1.27 years in the HFS group. The study was approved by the Taichung Veteran General Hospital Research Ethical Board in Taichung, Taiwan (CE16282B), and all participants provided written informed consent before participating in the study. All methods were performed in accordance with the Declaration of Helsinki guidelines and hospital regulations.

### Clinical Assessment

The QTT was performed in all subjects to measure warm and cold thresholds in 4 limbs using the PATHYWAY systems (Medoc, Ramat Yishay, Israel). A thermode of the device which could heat up or cool down from the baseline of 32°C was placed on the subject's skin. The subject then gave a response to the examiner when they felt the slightest temperature change (either warmer or colder), and the temperature was recorded. This was repeated three times each for warm and cold, and the mean temperatures were calculated as the warm and cold detection thresholds.

The patients with BSP were evaluated with the Jankovic Rating Scale (JRS) and the Blepharospasm disability index (BSDI). The JRS contains two subscales of severity and frequency, both of which are scored on a 5-point scales ranging from 0 to 4, with 4 indicating the most severe or frequent symptom. The BSDI represents the impairment of six daily activities related to BSP. Each activity is rated from 0 to 4, with 4 indicating that it is not possible to perform the activity due to disease (18).

The patients with HFS were evaluated using a 5-point rating scale for symptom severity and seven self-rating items (HFS-7) for health-related quality of life. The 5-point rating scale was scored from 0 to 4, with a higher score indicating more severe symptoms. The HFS-7 questionnaire contains seven items regarding activities of daily living, emotional well-being, and stigma. All items are self-rated from 0 (never) to 4 (always) (19).

In addition, all of the participants received the Mini-Mental State Examination (MMSE) to evaluate cognition, and the Beck Depression Inventory II (BDI-II) and Beck Anxiety Inventory (BAI) to assess mood status.

### MRI Data Acquisition

For the MRI examination, the patients wore earplugs and headsets before entering the scan room. Inappropriate items such as ferromagnetic materials and electronic devices were removed for safety. During the resting-state scan, the participant was instructed to remain awake with their eyes closed, lie still, and think of nothing. Illumination was kept low to improve patient comfort. The first four volumes were considered as dummy scans and discarded automatically by the scanner.

Data were collected on a Philips 3T scanner (Achieva, Philips Healthcare, Best, Netherlands). A gradient echo-planar sequence with axial orientation was used to collect functional data (TR = 2000 ms, TE = 30 ms, flip angle = 90°, 40 slices with 3 mm thickness and 1 mm gap, voxel size = 3 × 3 × 3 mm^3^. A high-resolution T1-weighted MPRAGE (TR = 8 ms, TE = 3.7 ms, flip angle = 15°, 168 slices, voxel size = 1.0 × 1.0 × 1.0 mm^3^) was obtained.

### MRI Data Analysis

Preprocessing of fMRI data was performed using Functional Connectivity Toolbox (CONN) pipeline version 18.b (www.nitrc.org/projects/conn, RRID:SCR_009550). First, slice-timing correction was performed. The slices were acquired in ascending, interleaved order, and the images were realigned to correct for subject head motion. Functional and structural segmentations were done to divide the data into different tissue classes: white matter, gray matter, and cerebrospinal fluid (CSF). The images were then normalized to a Montreal Neurological Institute template, and the normalized images were resliced with a target resolution of 2 mm. The normalized fMRI images were then smoothed with an 8-mm full width at half maximum Gaussian kernel. The motion outlier threshold of artifact detection tool was set at 95th percentiles in normative sample with motion threshold 0.5 mm. In the preprocessing pipeline, ART-based identification was applied, and acquisitions with displacement above the threshold were removed. Brain masking process was applied for voxel-level analysis. A band-pass filter from 0.01 Hz to infinity were used.

To investigate functional connectivity, we performed seed-to-voxel analysis to our acquiring data. For the seeds selection, we chose all pre-defined seeds of the CONN toolbox that belong to functional networks, including (1) Default Mode Network (4 ROIs), (2) Frontoparietal Network (4 ROIs), (3) Sensorimotor Network (3 ROIs), (4) Salience Network (7 ROIs), (5) Dorsal Attention Network (4 ROIs), (6) Visual Network (4 ROIs), (7) Language Network (4 ROIs), and (8) Cerebellar Network (2 ROIs) (Supplementary Figure S1). All results were displayed under the significance of *p* = 0.001 (uncorrected, peak-level) and *p* = 0.05 (false discovery rate (FDR) corrected, cluster-level). To compare the effects between the BSP patients and HCs and between the HFS patients and HCs, analysis of covariance (ANCOVA) was used to control for covariates (age and gender) in the CONN toolbox. Multiple regression analysis was used to analyze the unique effect of temperature threshold, which was also adjusted for age and gender.

### Other Statistical Analysis

Clinical characteristics between the groups were compared using the Kruskal-Wallis test as a non-parametric test for continuous variables, and the chi-square test for binary variables. All statistical analyses were performed using SPSS version 20 (IBM Corporation, Armonk, New York, USA), and statistical significance levels were set at *p* < 0.05.

## Results

### Demographic Information

There was a significant difference in cold detection threshold of the QTT over the lower extremities between the three groups (*p* = 0.044) (Table 1). Post hoc analysis revealed that the BSP and HFS groups had a higher cold detection threshold than the HC group, but there was no significant difference between the BSP and HFS groups. There were no significant differences in other QTT test parameters including warm detection threshold over the upper and lower extremities and cold detection threshold over the upper extremities. In addition, there were no significant differences in age, gender, disease duration, duration of BTX treatment, MMSE, BDI-II, or BAI between the three groups.

**Table 1:** Clinical characteristics of the study subjects.

	**Total**	**Control**	**BSP**	**HFS**	***P***
**Variable**	***n* = 38**	***n* = 15**	***n* = 12**	***n* = 11**	
Age (years old)	63.0 ± 9.5	60.5 ± 6.7	67.8 ± 6.6	61.2 ± 13.6	0.080
Gender (Male) %	14 (36.8)	4 (26.6)	4 (33.3)	6 (54.5)	0.331
MMSE	28.0 ± 2.0	28.8 ± 1.01	26.7 ± 2.8	28.4 ± 1.2	0.081
BDI-II	6.3 ± 5.8	5.4 ± 4.2	5.8 ± 5.0	8.0 ± 8.1	0.843
BAI	6.4 ± 6.0	6.1 ± 5.3	4.3 ± 4.8	9.0 ± 7.6	0.280
QTT warm (°C)					
Upper extremities	34.4 ± 2.2	34.0 ± 0.43	35.1 ± 3.9	34.4 ± 0.7	0.447
Lower extremities	41.1 ± 3.4	39.4 ± 2.8	42.1 ± 3.4	42.3 ± 3.4	0.051
QTT cold (°C)					
Upper extremities	29.8 ± 1.5	30.1 ± 0.5	29.5 ± 2.6	29.8 ± 0.5	0.306
Lower extremities	28.4 ± 2.0	29.1 ± 1.0	27.5 ± 2.8	28.3 ± 1.9	0.044
Disease duration (years)			8.3 ± 5.3	8.5 ± 3.6	0.929
Duration of BTX treatment (years)			3.8 ± 0.9	3.8 ± 1.2	0.833
Disease severity					
JRS-Severity			2.6 ± 0.4		
JRS-Frequency			2.8 ± 0.5		
Five-point RS				2.0 ± 0.0	
Disease disability					
BSDI			6.0 ± 4.0		
HFS-7				4.0 ± 3.7	

*Categorical data were analyzed using the chi-square test and are presented as number (%). Continuous data were analyzed using the Kruskal-Wallis test and are expressed as mean ± standard deviation (SD). P, p-value; BSP, blepharospasm; HFS, hemifacial spasm; MMSE, Mini Mental Status Examination; BDI-II, Beck Depression Inventory-II; BAI, Beck Anxiety Inventory; QTT, quantitative thermal test; BTX, botulinum toxin; JRS, Jankovic Rating Scale; Five-point RS, 5-point rating scale; BSDI, Blepharospasm Disability Index*.

### Resting-State fMRI, Seed-to-Voxel Functional Connectivity

**Figure 1:**
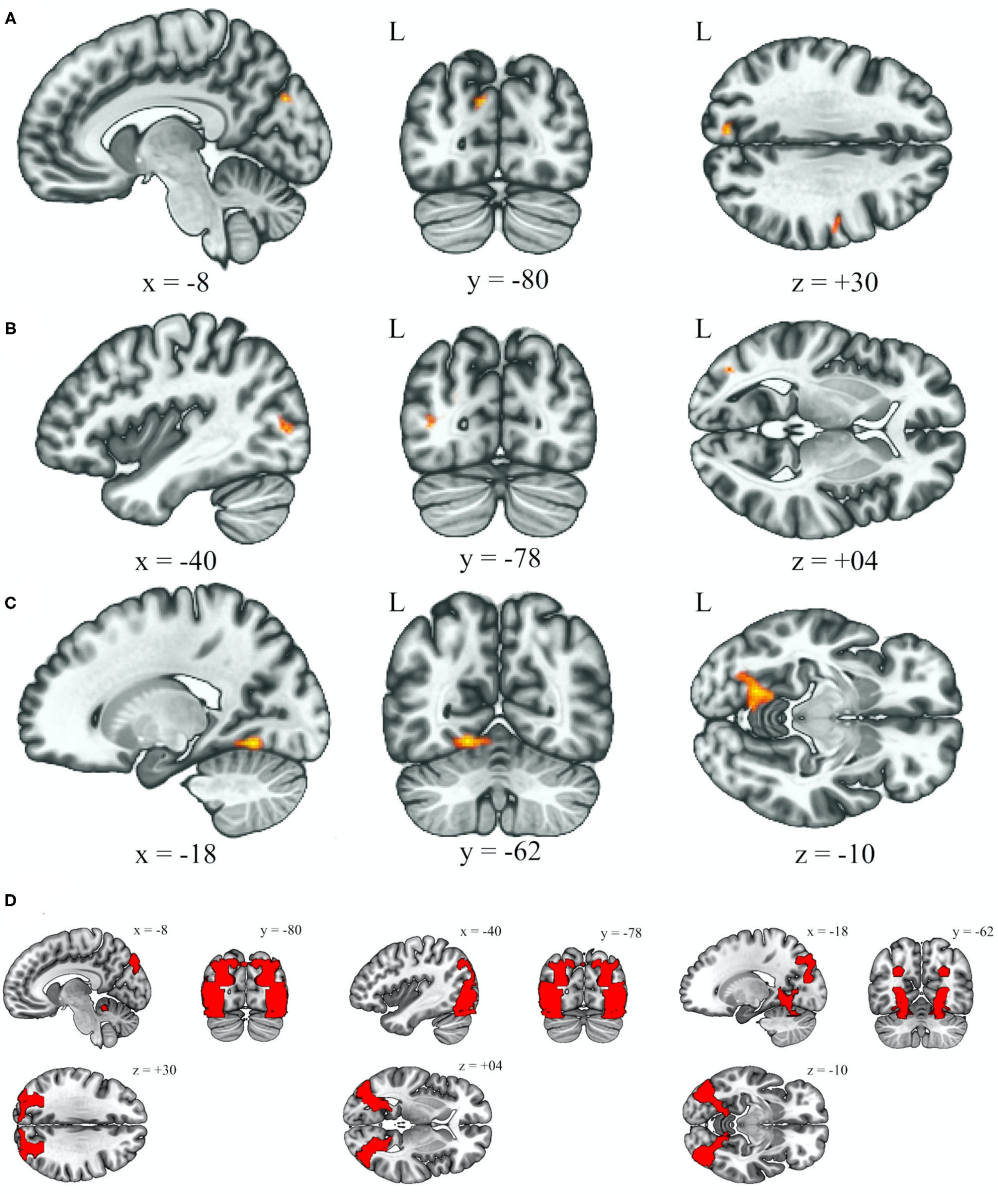
Increased functional connectivity with the anterior cerebellum as the seed ROI (*p* < 0.05, p-FDR-corrected) over the left occipital lobe (BA19), in the BSP group vs. HC group **(A)**, HFS group vs. HC group **(B)**, and the effect of cold detection threshold of the QTT over the lower extremities in all subjects **(C)**. Illustrations of BA19 in corresponding coordinates (x, y, z), left (−8, −80, +30), middle (−40, −78, +04), right (−18, −62, −10) **(D)**. L, left; ROI, region of interest; BA, Broadmann area; BSP, blepharospasm; HC, healthy control; HFS, hemifacial spasm; QTT, quantitative thermal test.

Table 2 reveals that the BSP group demonstrated an increased functional connectivity between the anterior cerebellar network and left cuneal cortex (Broadmann area 19, BA19) when comparing to the HC group after adjusting for age and gender (Figure 1A, left cuneal cortex, *p uncorrected* = 0.000026, *p FDR* = 0.000072). Increased functional connectivity between the anterior cerebellar network and left lateral occipital cortex (BA19) was found in the HFS group when comparing to the HC group after adjusting for age and gender (Figure 1B, lateral occipital cortex, *p uncorrected* = 0.000024, *p FDR* = 0.000024). Increased connectivity between the anterior cerebellar network and left lingual gyrus (BA19) was correlated with an impaired cold detection threshold over the lower extremities after adjusting for age and gender in all subjects (Figure 1C, *p uncorrected* = 0.000004, *p FDR* = 0.000004).

**Table 2:** Increased functional connectivity in different groups for seed ROI with the anterior cerebellar network.

**Groups**	**Target**	**Functional area**	**Peak MNI coordinate (x y z)**	**Cluster size (voxel)**	***P***
BSP vs. HC	Postcentral and precentral gyrus R	Primary somatosensory and motor cortex (BA 1–3, 4)	+56 −10 +32	139	0.0000
	Postcentral gyrus R	Primary somatosensory cortex (BA 1–3)	+16 −34 +64	119	0.0001
	Cuneal cortex L	Associative visual cortex (BA 19)	−8 −80 +30	119	0.0000
HFS vs. HC	Inferior and superior lateral occipital cortex L	Secondary and associative visual cortex (BA 18, 19)	−40 −78 +04	165	0.0000
Effect of QTT scores (lower extremities)	Lingual gyrus, Occipital fusiform gyrus, Cerebellum 4 and 5, Cerebellum 6 L	Secondary and associative visual cortex (BA 18, 19)	−18 −62 −10	387	0.0000

*Negative coordinates are left (x), posterior (y), inferior (z). P, p-value; MNI, Montreal Neurological Institute; ROI, region of interest; BSP, blepharospasm; HC, healthy control; HFS, hemifacial spasm; QTT, quantitative thermal test; R, Right; L, Left; BA, Brodmann area*.

**Figure 2:**
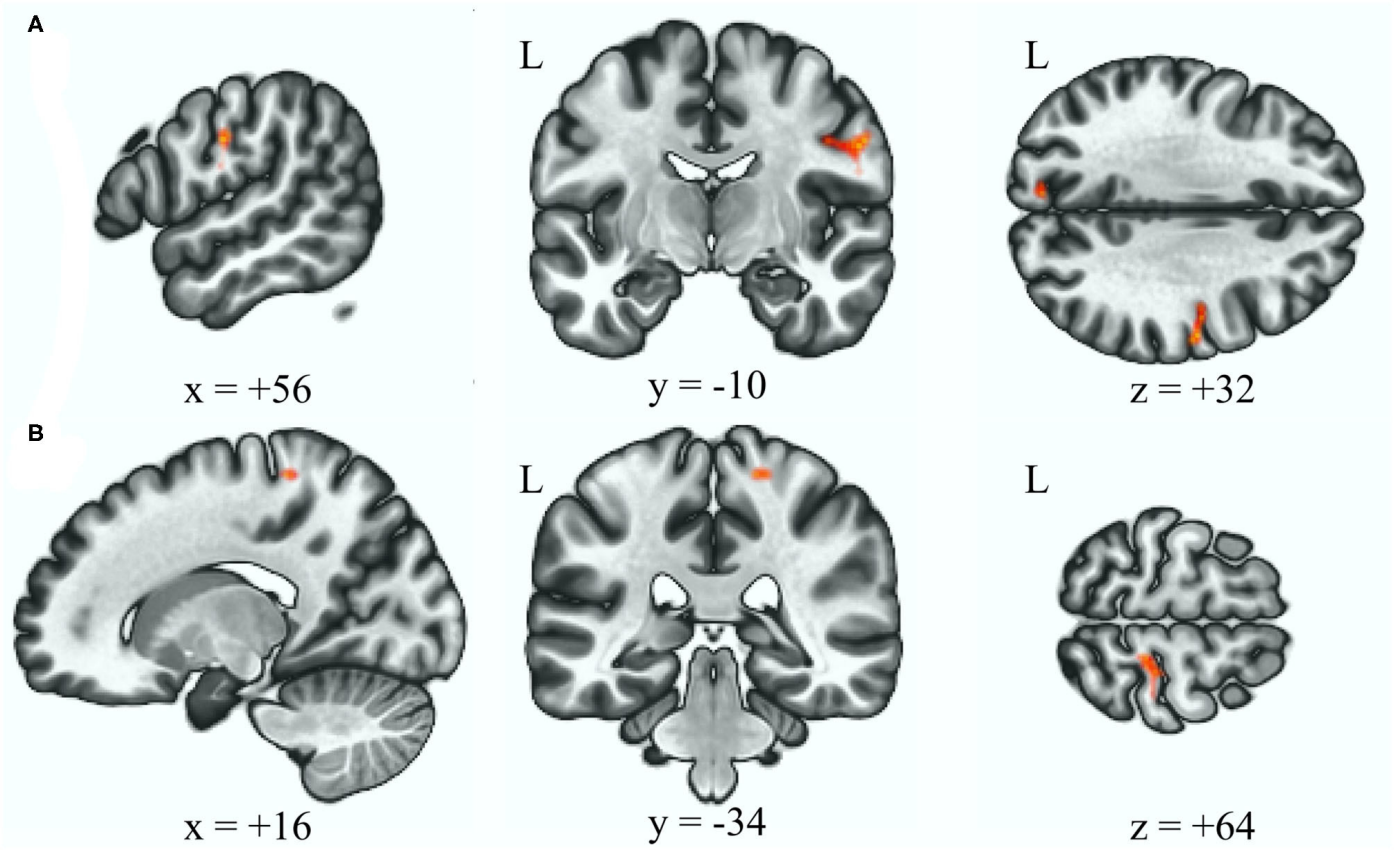
Increased functional connectivity with the anterior cerebellum as the seed ROI (*p* < 0.05, p-FDR-corrected) in the BSP group vs. HC group, over the right postcentral and precentral gyrus **(A)**, and over the right postcentral gyrus **(B)**. L, left; ROI, region of interest; BSP, blepharospasm; HC, healthy control.

Table 2 reveals that the BSP group demonstrated an increased connectivity between the anterior cerebellar network and right precentral and postcentral gyri after adjusting for age and gender relative to the HC group (Figure 2, *p uncorrected* = 0.000048, *p FDR* = 0.000072, and *p uncorrected* = 0.00012, *p FDR* = 0.00012, respectively).

**Figure 3:**
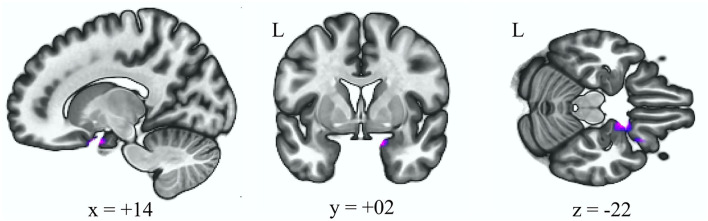
Decreased functional connectivity with the anterior cerebellum as the seed ROI (*p* < 0.05, p-FDR-corrected) in the HFS group vs. HC group. L, left; ROI, region of interest; HFS, hemifacial spasm; HC, healthy control.

Table 3 reveals that there was reduced connectivity between the anterior cerebellar network and right frontal orbital cortex after adjusting for age and gender in the HFS group when comparing to the HC group (Figure 3, *p uncorrected* < 0.000001, *p FDR* < 0.000001).

**Table 3:** Decreased functional connectivity in different groups for seed ROI with the anterior cerebellar network.

**Groups**	**Target**	**Functional area**	**Peak MNI coordinate (x y z)**	**Cluster size (voxel)**	***P***
HFS vs. HC	Frontal orbital cortex, amygdala, Parahippocampal gyrus R	Orbitofrontal and subgenual area (BA 11, 25), dorsal entorhinal cortex (BA 34)	+14 +02 −22	340	0.0000

*Negative coordinates are left (x), posterior (y), inferior (z). P, p value; MNI, Montreal Neurological Institute; ROI, region of interest; HC, healthy control; HFS, hemifacial spasm; R, Right; BA, Brodmann area*.

## Discussion

This study suggests that the cerebellum plays an role in the central network disturbance in BSP and HFS. Increased rsFC between the anterior cerebellar network and left occipital regions (BA19) was found in both BSP and HFS groups (Figures 1A,B,D). Increasing evidence suggests that network deficits in the cerebellum, thalamus, and cortex result in altered sensorimotor integration, leading to BSP (2, 20, 21). Although HFS is mostly caused by peripheral irritation, a previous study using structural MRI reported reduced local connectivity in the cerebellum in both HFS and BSP patients (13). Moreover, our data showed that there was increased rsFC between the anterior cerebellar network and left lingual lobe (BA19), which correlated with impaired cold detection threshold over lower extremities in all subjects (Figures 1C,D). This finding may support the perspective that the cerebellum acts as a center of clusters processing proprioception, discrimination, and somatosensory thresholds, and it also underlies non-nociceptive processes such as attention and decision-making which predicts the pain (20, 22).

In this study, cold threshold test showed that the BSP and HFS patients were more insensitive to coldness in lower limbs than the HC group. This suggested that altered somatosensory processing may play a role in both BSP and HSP. The pathophysiology of dystonia involves defects in sensory or perceptual processing (2). Most previous studies using functional imaging have reported altered activity in the sensorimotor cortex (SMC) in patients with dystonia (23, 24). One study of patients with BSP reported increased activity over the SMC when whistling (6). Another resting fMRI study also reported decreased rsFC in the SMC to several seeds (7). Our data revealed that impaired cold threshold detection in lower extremities was associated with increased rsFC between left lingual lobe and anterior cerebellar network, and therefore supports the view that lingual gyrus may be involved in somatosensory stimulation and perception of sensory stimuli (25). In addition, the occipital lobe has been reported to be a pain processing brain network in previous studies (22, 26–28). One study investigating pain sensitivity with pain-free resting-state functional brain connectivity also suggested that several connections involving the occipital lobe influence the prediction of pain sensitivity (29). These findings suggest that dealing with the sensory information is possibly one of the functions of the occipital lobe. When comparing to the control group, both the BSP and HFS groups has increased rsFC between the left occipital regions (BA19) and anterior cerebellar network. We suppose that BA19, being part of occipital lobe and composed of the lingual gyrus, cuneus, and lateral occipital gyrus, may have its functional role in BSP, HFS and sensory processing. Considering with the interconnection within the occipital lobe (25, 30), altered sensory integration in the occipital lobe might be one of the plausible explanations for the rsFC within BA19 and impaired thermal detection in both BSP and HFS. Clinically BSP can result in functional blindness. The converted occipital function is probably related to the interruption of visual inputs, as glucose hypometabolism in the visual cortex has been observed in BSP (7, 31). Alternatively, activation of the visual cortex in BSP may suggest that the visuomotor pathway mediates facial hyperkinesia such as blinking to visual stimuli (32). Nevertheless, altered functional connectivity of the cerebellar visual network has also been shown in patients with other focal task-specific dystonia such as writer's cramp or embouchure dystonia which do not involving blinking (33).

In this study, some differences in the altered rsFC were revealed between the BSP and HFS groups. With the cerebellum as the seed ROI, the BSP group showed increased rsFC in the somatosensory and motor cortex, while the HSP showed decreased rsFC in the orbitofrontal cortex and amygdala. Changes in the activation level in the sensorimotor cortex have generally been reported in dystonia, including BSP (2, 20). Previous studies on resting state fMRI have shown decreased rsFC in the sensorimotor cortex within the sensorimotor network (34). Compared to the dysfunction of integration in the sensorimotor network, increased rsFC between the cerebellar network and sensorimotor cortex may explain the cerebellar modulation of sensorimotor plasticity (35). An atrophic amygdala together with increased rsFC of the amygdala to orbitofrontal cortex, medial prefrontal cortex, and insula have been reported in HFS patient (11). These abnormalities may lead to the emotional and visual deficiency, since the amygdala coordinates efferent and afferent information such as pain, anxiety, fear, reward, and visual information (36). In our study, although no difference in BAI was noted between the HSP and HFS groups, we speculate that the changes in rsFC between the cerebellar seed ROI, orbital frontal cortex, and amygdala may be a hint of the altered visuomotor or sensory processing in HFS.

There are several limitations to this study. First, although there were some statistically significant results in this study, our sample size was relatively small. Second, the information obtained from resting state functional images may not explain the causal relationship between clinical symptoms and alteration of brain functional communication Third, this study was a cross-sectional design, and we were unable to observe alterations of functional state with disease progression over time. Further studies with more subjects, a longitudinal design, or using multi-modalities should be performed.

In conclusion, the BSP and HFS patients have impaired cold detection threshold in lower extremities. Altered functional connectivity between the anterior cerebellar network and left occipital regions, especially the BA19, may be associated with BSP, HFS, and impaired cold detection threshold. The cerebellum and occipital lobe might have their functional role in BSP and HFS, and we postulate that impaired somatosensory processing may be one of the possible pathophysiological mechanisms. In addition, the differences in the rsFC to the anterior cerebellar network seed, including increased rsFC within the sensorimotor cortex in the BSP patients and decreased rsFC within the orbitofrontal cortex and amygdala in the HFS patients, may indicate clinical relevance to the disease entity. We believe that these findings provide a unique insight, and may help to understand and distinguish the pathophysiology between BSP and HFS in the future.

## Data Availability Statement

The raw data supporting the conclusions of this article will be made available by the authors, without undue reservation.

## Ethics Statement

The studies involving human participants were reviewed and approved by Taichung Veteran General Hospital Research Ethical Board in Taichung, Taiwan. The patients/participants provided their written informed consent to participate in this study.

## Author Contributions

T-CF, C-MC, and Y-JG conceptualized the project. M-HC and C-HW performed the data acquisition and investigation. T-CF wrote the first draft of the manuscript. Y-JG critically reviewed the manuscript. All authors contributed to writing and revising the manuscript.

## Funding

This work was supported by Taichung Veterans General Hospital [grant numbers 106DHA0500161].

## Conflict of Interest

The authors declare that the research was conducted in the absence of any commercial or financial relationships that could be construed as a potential conflict of interest.

## Publisher's Note

All claims expressed in this article are solely those of the authors and do not necessarily represent those of their affiliated organizations, or those of the publisher, the editors and the reviewers. Any product that may be evaluated in this article, or claim that may be made by its manufacturer, is not guaranteed or endorsed by the publisher.
