# Supplementary material for: Altered Functional Connectivity and Sensory Processing in Blepharospasm and Hemifacial Spasm: Coexistence and Difference
Source: Front Neurol. 2021 Dec 15;12:759869. doi: 10.3389/fneur.2021.759869 (PMC8715087; doi:10.3389/fneur.2021.759869)
Supplement: Supplementary file 1 [file Table_1.DOCX]

| 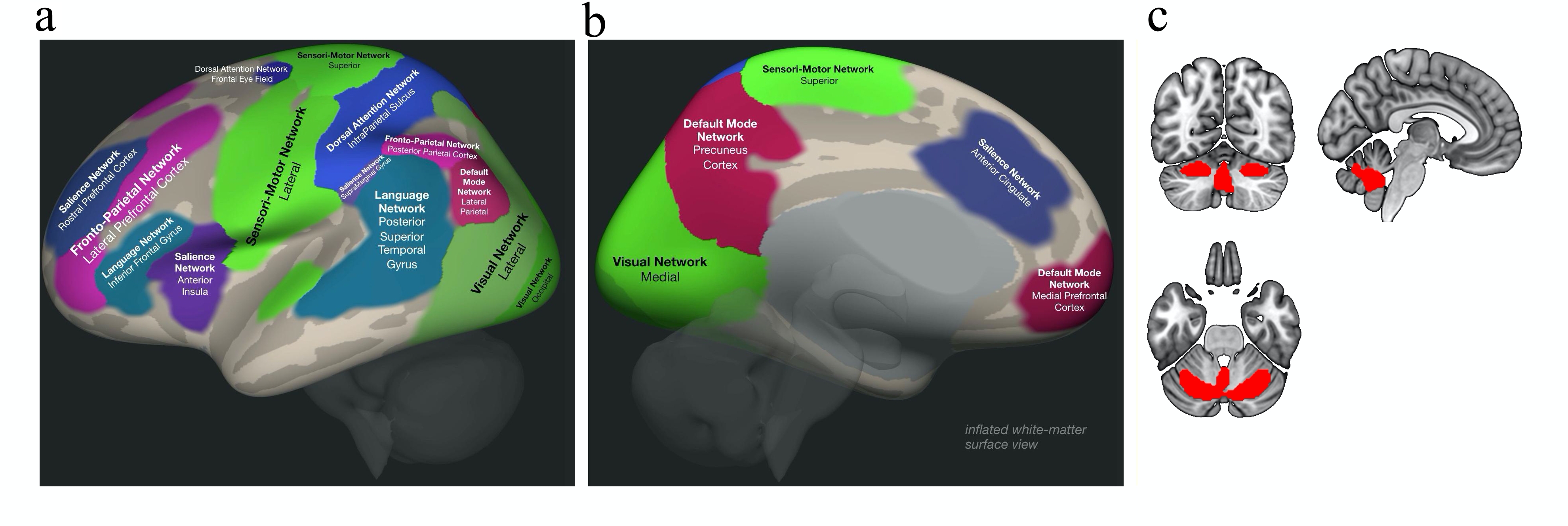 |
| --- |
| Figure S1. Illustration and description of pre-defined networks in the CONN toolbox (a, b). Illustration of anterior cerebellar network which is defined in the CONN toolbox (c).  *Note.* Figure S1a and S1b are adapted from the CONN toolbox version 18.b (https://web.conn-toolbox.org). |
